# Development of a prognostic model based on nutritional and inflammatory indicators for predicting postoperative survival in esophageal cancer: a retrospective study

**DOI:** 10.3389/fimmu.2026.1701862

**Published:** 2026-04-14

**Authors:** Huike Wang, Zhe Wang, Bingtong Yue, Xi Luo, Yalan Yang, Yu Chen, Feng Wang

**Affiliations:** 1 Department of Oncology, The First Affiliated Hospital of Zhengzhou University, Zhengzhou, Henan, China; 2 Tianjian Laboratory for Advanced Biomedical Sciences, Zhengzhou, Henan, China; 3 State Key Laboratory of Metabolic Dysregulation & the Prevention and Treatment of Esophageal Cancer, Zhengzhou, Henan, China

**Keywords:** esophageal cancer, inflammation–immune–nutrition score, nomogram, prognostic model, restricted cubic spline

## Abstract

**Introduction:**

Systemic inflammation, immunity, and nutritional status are integral to tumor biology, shaping the microenvironment and influencing esophageal cancer (EC) outcomes. Yet, their integration into pragmatic prognostic tools—and potential implications for immunotherapy stratification—remain limited. This retrospective study assessed the prognostic value of the inflammation–immunity–nutrition score (IINS) and red cell distribution width-to-lymphocyte ratio (RLR), indicators reflecting host immunity, systemic inflammation, and nutritional reserve, in EC patients.

**Methods:**

Clinical data from 660 EC patients who underwent radical surgery (2012–2018) were retrospectively analyzed and randomly assigned to training (*n* = 459) and validation (*n* = 201) cohorts. Candidate predictors were screened using LASSO and entered into multivariable Cox models. A nomogram incorporating IINS, RLR, and clinical covariates was constructed and validated with the C-index, calibration, and time-dependent AUC; clinical utility was evaluated with decision curve analysis (DCA), Integrated Discrimination Improvement (IDI), and Net Reclassification Index (NRI).

**Results:**

IINS, RLR, and eight additional factors were independent prognostic variables. The nomogram showed good calibration and superior discrimination versus AJCC staging, with a higher C-index and AUC in both cohorts. DCA, IDI, and NRI confirmed greater net benefit and improved risk reclassification.

**Conclusions:**

This study proposes and internally validates a nomogram linking immune–nutritional surrogates with survival in EC. By reflecting systemic inflammation and host immunity, the model supports individualized risk stratification, perioperative optimization, and may inform patient selection for immunotherapy. External multicenter validation is warranted.

## 1 Introduction

Esophageal cancer (EC) is a common malignant tumor of the digestive tract with high morbidity and mortality (1–3). Despite advances in multimodal therapy, long-term survival outcomes of EC remain highly heterogeneous. Radical surgery (RS) is the main treatment method for early EC. Recent therapeutic advances have improved survival after RS. Therefore, improved prognostic stratification tools are essential for individualized risk assessment in EC patients.

The American Joint Committee on Cancer (AJCC) tumor-node-metastasis (TNM) staging system is the standard framework for prognostic assessment and clinical decision-making in esophageal cancer. However, substantial survival heterogeneity persists among patients with the same TNM stage, indicating that TNM classification alone may be insufficient for individualized prognosis (4, 5). Moreover, nomograms provide individualized risk estimation by integrating multiple prognostic variables into a quantitative and visual tool. Compared with TNM staging alone, nomogram-based models may improve risk stratification and support personalized postoperative management (6–8).

Systemic inflammatory and nutritional indices derived from routine blood tests, such as the neutrophil-to-lymphocyte ratio (NLR), platelet-to-lymphocyte ratio (PLR), and prognostic nutritional index (PNI), have been reported to correlate with survival in multiple malignancies, including esophageal cancer (9, 10). These markers are thought to reflect the balance between tumor-promoting inflammation and host immune response. However, most studies have evaluated these indicators individually, and few have systematically integrated immune, inflammatory, and nutritional parameters into a unified prognostic model specifically for EC patients undergoing radical surgery. Therefore, whether combined immune–inflammatory–nutritional indices provide incremental prognostic value beyond traditional staging remains unclear (11–20).

This study investigated the risk factors for EC patients after RS and quantified them by constructing a nomogram model based on inflammation–immunity–nutrition score (IINS), red cell distribution width-to-lymphocyte ratio (RLR), and routine clinical examination parameters, providing a basis for formulating a more reasonable and accurate individualized treatment plan.

## 2 Methods

### 2.1 Study design and cohort selection

**Figure 1:**
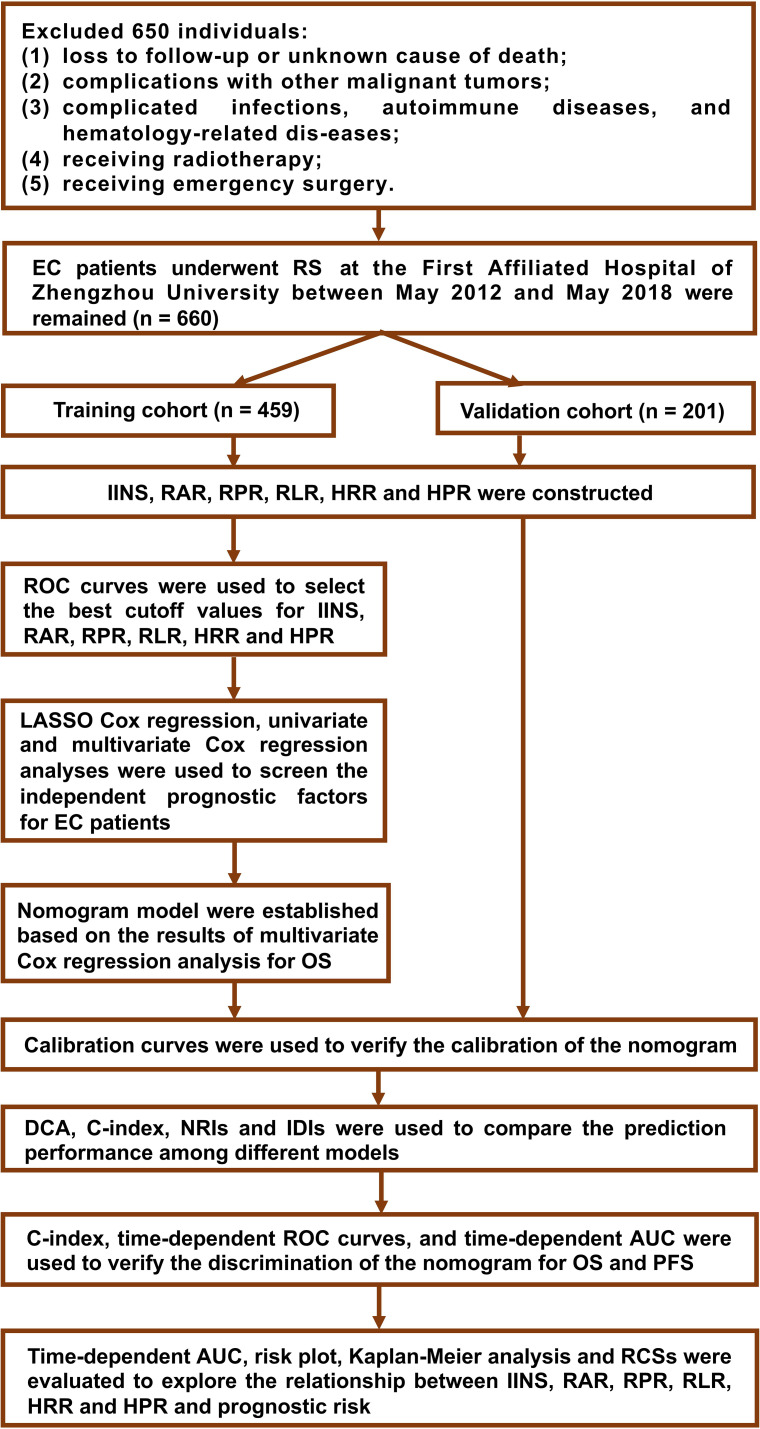
Flow chart of the study design.

The preoperative and postoperative clinicopathological data of EC patients in the First Affiliated Hospital of Zhengzhou University from May 2012 to May 2018 were retrospectively collected. All patients received RS therapy. Patients were randomly divided into a training cohort (459 cases) and a validation cohort (201 cases) in a seven-to-three ratio. **Figure 1** shows the study design process.

The inclusion criteria were as follows: (1) pathological confirmation of esophageal cancer; (2) radical surgery performed at our institution; and (3) availability of complete clinicopathological and laboratory data. The exclusion criteria were as follows: (1) loss to follow-up or unknown cause of death; (2) history of other malignant tumors; (3) presence of active infection, autoimmune disorders, or hematologic diseases; (4) receipt of radiotherapy; and (5) emergency surgery. Neoadjuvant therapy and combination therapy in this study referred exclusively to chemotherapy-based regimens without any radiotherapy component. These criteria were applied to minimize nontumor-related systemic inflammatory interference and to ensure data integrity for prognostic modeling.

### 2.2 Outcomes and follow-up

The follow-up of patients followed the guidelines for the diagnosis and treatment of EC: 1 ~ 2 years after RS: review every 3 ~ 6 months; 3 ~ 5 years: review every 6 months; and after 5 years, review annually. Follow-up included (1) medical history and physical examination; (2) upper digestive tract angiography; (3) chest and abdominal enhanced CT scans; (4) cervical hyperplasia; and (5) endoscopy. The primary endpoint of this study was OS, and the secondary endpoint was PFS. OS was defined as the time from radical surgery to death from any cause. PFS was defined as the time from radical surgery to disease progression or death, whichever occurred first. Follow-up continued until May 2022. OS is defined as the time from randomization to death from any cause. PFS is defined as the time from randomization to disease progression or death from any cause. RS was defined as complete resection without microscopic residual cancer. Tumor size was defined as the maximum diameter of postoperative gross pathology. For organizational types, refer to the WHO classification of the digestive system, 2019 edition. The staging was performed according to the AJCC TNM Staging, Seventh Edition.

### 2.3 Definition

In the training cohort, by subtracting the maximum value of the *x*-axis from the *y*-axis of the ROC curve (i.e., Youden index), the optimal diagnostic critical value of CRP, ALB, LYM, and other detection indicators, namely, the cutoff value, was determined to obtain better sensitivity and specificity (**Table 1**). The IINS was constructed by dichotomizing each component according to cohort-specific optimal cutoff values, with each parameter assigned a score of 0 or 1. The total IINS ranged from 0 to 3, with higher scores indicating poorer immune–nutritional status. In addition, the detailed calculation method of IINS is shown in **Table 2**. We divided IINS into a low IINS score group (0 or 1 point) and a high IINS score group (2 or 3 points).

**Table 1:** Diagnostic value of variables.

Variables	Cutoff value	Sensitivity	Specificity	AUC (95% CI)	*p*-value
IINS	1.500	0.619	0.962	0.790 (0.741 ~ 0.840)	< 0.001
RAR	0.310	0.609	0.923	0.765 (0.708 ~ 0.822)	< 0.001
RPR	0.082	0.219	0.519	0.644 (0.563 ~ 0.724)	0.001
RLR	7.413	0.580	1.000	0.790 (0.745 ~ 0.835)	< 0.001
HRR	9.306	0.904	0.533	0.684 (0.624 ~ 0.745)	< 0.001
HPR	0.625	0.904	0.560	0.726 (0.666 ~ 0.786)	< 0.001
ALB (g/L)	42.150	0.923	0.595	0.772 (0.718 ~ 0.826)	< 0.001
CRP (mg/L)	0.910	0.870	0.654	0.816 (0.757 ~ 0.874)	< 0.001
LYM (× 10^9^/L)	1.790	1.000	0.568	0.821 (0.778 ~ 0.863)	< 0.001
RBC (× 10^12^/L)	4.065	0.962	0.516	0.714 (0.657 ~ 0.771)	< 0.001
Hct (L/L)	0.358	0.641	0.981	0.863 (0.825 ~ 0.901)	< 0.001
WBC (× 10^9^/L)	6.305	0.373	0.712	0.512 (0.431 ~ 0.593)	0.778
Neut (× 10^9^/L)	3.215	0.631	0.942	0.801 (0.755 ~ 0.848)	< 0.001
Mono (× 10^9^/L)	0.405	0.646	0.115	0.790 (0.743 ~ 0.836)	< 0.001
PDW (fL)	14.900	0.585	1.000	0.766 (0.720 ~ 0.812)	< 0.001
Pct (%)	0.216	0.322	0.865	0.573 (0.495 ~ 0.651)	0.086
MPV (fL)	9.750	0.319	0.173	0.576 (0.494 ~ 0.659)	0.072
TP (g/L)	65.850	0.415	0.981	0.772 (0.726 ~ 0.818)	< 0.001
GLOB (g/L)	24.250	0.641	0.962	0.797 (0.755 ~ 0.839)	< 0.001
PA (mg/L)	208.500	0.575	0.808	0.707 (0.638 ~ 0.777)	< 0.001
LDL (mmol/L)	2.535	0.619	0.923	0.751 (0.700 ~ 0.803)	< 0.001
T-CHO (mg/dL)	4.325	1.000	0.575	0.841 (0.796 ~ 0.886)	< 0.001
Fib (g/L)	3.085	0.614	0.981	0.824 (0.781 ~ 0.867)	< 0.001
PCT (ng/mL)	0.022	0.907	0.788	0.770 (0.677 ~ 0.863)	< 0.001
TAP (µm^2^)	114.048	0.786	1.000	0.859 (0.826 ~ 0.891)	< 0.001

**Table 2:** IINS scoring criteria.

Variables	Low value	High value
ALB (g/L)	< 42.150	≥ 42.150
Score	1	0
CPR (mg/L)	< 0.910	≥ 0.910
Score	0	1
LYM (× 10^9^/L)	< 1.790	≥ 1.790
Score	1	0

IINS = CRP Score (0/1) + LYM Score (0/1) + ALB Score (0/1).

In addition, we calculated inflammation and nutritional markers based on the preoperative blood counts: RAR = red blood cell distribution width (RDW)/albumin (ALB), RPR = RDW/platelet (PLT), HRR = hemoglobin (Hb)/RDW, HPR = Hb/PLT, and RLR = RDW/lymphocyte (LYM). Plateletcrit (Pct, %) and procalcitonin (PCT, ng/mL) were included as candidate laboratory variables.

### 2.4 Statistical analysis

Taking the patient cohort of the First Affiliated Hospital of Zhengzhou University as the study cohort, a nomogram including IINS and RLR was constructed, and the model was internally verified in the cohort. ROC and the maximum value of the Youden index (Youden index = sensitivity + specificity − 1) were used to determine the optimal threshold for predicting OS. Optimal cutoff values were determined using ROC curve analysis with the Youden index to maximize sensitivity and specificity. The Chi-square test was used to analyze the significant differences between different cohorts and clinicopathologic features, as well as nutritional/inflammatory indicators. All statistical analyses were performed using SPSS software (version 26.0) and RStudio software (version 4.2.1). Alpha = 0.05 (two-tailed). Missing data were minimal and handled using a complete-case approach. The overall missing rate was below 5%; therefore, no imputation or sensitivity analyses were performed. Stepwise selection was applied only as a complementary method and not as the primary variable filtering strategy.

LASSO Cox regression was first applied to all candidate inflammatory and nutritional variables to reduce multicollinearity and identify the most informative predictors associated with survival outcomes. Variables retained by the LASSO model were subsequently evaluated using univariate Cox proportional hazards regression. Factors with *p* < 0.10 were further incorporated into multivariable Cox regression models to determine independent prognostic predictors. To ensure model robustness, multivariable Cox regression was performed using forward, backward, and stepwise selection strategies, and model performance was compared based on AIC values using analysis of variance (ANOVA). The final prognostic model was constructed using variables that consistently remained significant in multivariable analyses (*p* < 0.05).

Subsequently, the internal validation of the nomogram and the comparison between the nomogram and the AJCC staging system were carried out. The C-index, time-dependent ROC, and time-dependent AUC were used to evaluate the prediction accuracy of the model. The evaluation criteria were as follows: good (AUC/C-index greater than 0.8), medium (0.7 greater than or equal to AUC/C-index less than or equal to 0.8), or poor (AUC/C-index less than 0.8). Calibration curves were used to evaluate the difference between the predicted outcome time of the model and the actual outcome time. Decision curve analysis (DCA), NRI, and IDI assessed the clinical net benefit of our model compared to AJCC TNM staging alone.

Kaplan–Meier analysis and the log-rank test were used to verify the linear correlation between the levels of nutrition and inflammation indicators and OS/PFS. RCS analysis was used to verify the nonlinear relationship between these indicators and the hazard ratio (HR) of death.

## 3 Results

### 3.1 Study population characteristics

A total of 660 EC patients were randomly divided into a training cohort (459 cases) and a validation cohort (201 cases) at a ratio of seven to three. During a median follow-up of 84.1 months (calculated using the reverse Kaplan–Meier method), 588 death events (89.1%) were observed in the overall cohort. The remaining 72 patients (10.9%) were censored at the last follow-up date or due to loss to follow-up. The median OS was 25.23 months, and the median PFS was 18.57 months.

According to the demographic characteristics, clinicopathologic characteristics, and laboratory indicators of different cohorts (**Table 3**), the patients were mainly male < 65 years old, did not receive neoadjuvant therapy, had no complications, received chemotherapy, grade II, Tis+T1+T2, N0+N1, M0, lower esophageal segment, without vascular invasion or nerve invasion, and tumor size ≥3 cm. There were no significant differences between the two cohorts, so the results were comparable (*P* > 0.05).

**Table 3:** Demographic and clinical characteristics of patients with EC (*n* = 660).

Variables	Validation cohort (cases, %)	Training cohort (cases, %)	Whole population (cases, %)	*p*-value
Age (year)				0.290
< 65	121 (60.2%)	256 (55.8%)	337 (57.1%)	
≥ 65	80 (39.8%)	203 (30.8%)	283 (42.9%)	
Sex				0.239
Female	72 (35.8%)	143 (31.2%)	215 (32.6%)	
Male	129 (64.2%)	316 (68.8%)	445 (67.4%)	
Neoadjuvant therapy				0.782
No	160 (79.6%)	361 (78.6%)	521 (78.9%)	
Yes	41 (20.4%)	98 (21.4%)	139 (21.1%)	
Comorbidity				0.911
No	141 (70.1%)	320 (69.7%)	461 (69.8%)	
Yes	60 (29.9%)	139 (30.3%)	199 (30.2%)	
Treatment methods				0.048
NC	38 (18.9%)	54 (11.8%)	92 (13.9%)	
Chemotherapy	121 (60.2%)	307 (66.9%)	428 (64.8%)	
Combination therapy	42 (20.9%)	98 (21.4%)	140 (21.2%)	
Histologic subtypes				0.753
ESCC	190 (94.5%)	431 (93.9%)	621 (94.1%)	
Others	11 (5.5%)	28 (6.1%)	39 (5.9%)	
Histologic grade				0.546
I	45 (22.4%)	99 (21.6%)	144 (21.8%)	
II	109 (54.2%)	234 (51.0%)	343 (52.0%)
III	47 (23.4%)	126 (27.5%)	173 (26.2%)	
T stage				0.850
Tis + T1 + T2	107 (53.2%)	248 (54.0%)	355 (53.8%)	
T3 + T4	94 (46.8%)	211 (46.0%)	305 (46.2%)	
N stage				0.544
N0 + N1	174 (86.6%)	389 (84.7%)	563 (85.3%)	
N2 + N3	27 (13.4%)	70 (15.3%)	97 (14.7%)	
M stage				0.913
M0	199 (99.0%)	454 (98.9%)	653 (98.9%)	
M1	2 (1.0%)	5 (1.1%)	7 (1.1%)	
Tumor location				0.318
Upper	35 (17.4%)	89 (19.4%)	124 (18.8%)	
Middle	77 (38.3%)	148 (32.2%)	225 (34.1%)	
Lower	89 (44.3%)	222 (48.4%)	311 (47.1%)	
Vascular invasion				0.915
No	161 (80.1%)	366 (79.7%)	527 (79.8%)	
Yes	40 (19.9%)	93 (20.3%)	133 (20.2%)	
Nerve invasion				0.548
No	168 (83.6%)	392 (85.4%)	560 (84.8%)	
Yes	33 (16.4%)	67 (14.6%)	100 (15.2%)	
Tumor size (cm)				0.289
< 3	93 (46.3%)	192 (41.8%)	285 (43.2%)	
≥ 3	108 (53.7%)	267 (58.2%)	375 (56.8%)	
IINS				0.469
0	25 (12.4%)	78 (17.0%)	103 (15.6%)	
1	63 (31.3%)	127 (27.7%)	190 (28.8%)	
2	18 (9.0%)	40 (8.7%)	58 (8.8%)	
3	95 (47.3%)	214 (46.6%)	309 (46.8%)	
RAR				0.914
< 0.310	92 (45.8%)	208 (45.3%)	300 (45.5%)	
≥ 0.310	109 (54.2%)	251 (54.7%)	360 (54.5%)	
RPR				0.619
< 0.082	153 (76.1%)	341 (74.3%)	494 (74.8%)	
≥ 0.082	48 (23.9%)	118 (25.7%)	166 (25.2%)	
RLR				0.967
< 7.413	98 (48.8%)	223 (48.6%)	321 (48.6%)	
≥ 7.413	103 (51.2%)	236 (51.4%)	339 (51.4%)	
HRR				0.980
< 9.306	97 (48.3%)	222 (48.4%)	319 (48.3%)	
≥ 9.306	104 (51.7%)	237 (51.6%)	341 (51.7%)	
HPR				0.997
< 0.625	102 (50.7%)	233 (50.8%)	335 (50.8%)	
≥ 0.625	99 (49.3%)	226 (49.2%)	325 (49.2%)	
RBC (× 10^12^/L)				0.161
< 4.065	81 (40.3%)	212 (46.2%)	293 (44.4%)	
≥ 4.065	120 (59.7%)	247 (53.8%)	367 (55.6%)	
Hct (L/L)				0.658
< 0.358	90 (44.8%)	197 (42.9%)	287 (43.5%)	
≥ 0.358	111 (55.2%)	262 (57.1%)	373 (56.5%)	
WBC (× 10^9^/L)				0.890
< 6.305	129 (64.2%)	292 (63.6%)	421 (63.8%)	
≥ 6.305	72 (35.8%)	167 (36.4%)	239 (36.2%)	
Neut (× 10^9^/L)				0.799
< 3.215	85 (42.3%)	199 (43.4%)	284 (43.0%)	
≥ 3.215	116 (57.7%)	260 (56.6%)	376 (57.0%)	
Mono (× 10^9^/L)				0.830
< 0.405	85 (42.3%)	190 (41.4%)	275 (41.7%)	
≥ 0.405	116 (57.7%)	269 (58.6%)	385 (58.3%)	
PDW (fL)				0.979
< 14.900	97 (48.3%)	221 (48.1%)	318 (48.2%)	
≥ 14.900	104 (51.7%)	238 (51.9%)	342 (51.8%)	
Pct (%)				0.567
< 0.216	145 (72.1%)	321 (69.9%)	466 (70.6%)	
≥ 0.216	56 (27.9%)	138 (30.1%)	194 (29.4%)	
MPV (fL)				0.200
< 9.750	150 (74.6%)	320 (69.7%)	470 (71.2%)	
≥ 9.750	51 (25.4%)	139 (30.3%)	190 (28.8%)	
TP (g/L)				0.845
< 65.850	103 (51.2%)	239 (52.1%)	342 (51.8%)	
≥ 65.850	98 (48.8%)	220 (47.9%)	318 (48.2%)	
GLOB (g/L)				0.186
< 24.250	97 (48.3%)	196 (42.7%)	293 (44.4%)	
≥ 24.250	104 (51.7%)	263 (57.3%)	367 (55.6%)	
PA (mg/L)				0.151
< 208.500	82 (40.8%)	215 (46.8%)	297 (45.0%)	
≥ 208.500	119 (59.2%)	244 (53.2%)	363 (55.0%)	
LDL (mmol/L)				0.233
< 2.535	99 (49.3%)	203 (44.2%)	302 (45.8%)	
≥ 2.535	102 (50.7%)	256 (55.8%)	358 (54.2%)	
T-CHO (mg/dL)				0.683
< 4.325	99 (49.3%)	234 (51.0%)	333 (50.5%)	
≥ 4.325	102 (50.7%)	225 (49.0%)	327 (49.5%)	
Fib (g/L)				0.821
< 3.085	93 (46.3%)	208 (45.3%)	301 (45.6%)	
≥ 3.085	108 (53.7%)	251 (54.7%)	359 (54.4%)	
PCT (ng/mL)				0.167
< 0.022	26 (12.9%)	79 (17.2%)	105 (15.9%)	
≥ 0.022	175 (87.1%)	380 (82.8%)	555 (84.1%)	
TAP (µm^2^)				0.514
< 114.048	66 (32.8%)	139 (30.3%)	205 (31.1%)	
≥ 114.048	135 (67.2%)	320 (69.7%)	455 (68.9%)

### 3.2 Univariate and multivariate Cox regression analyses

**Figure 2:**
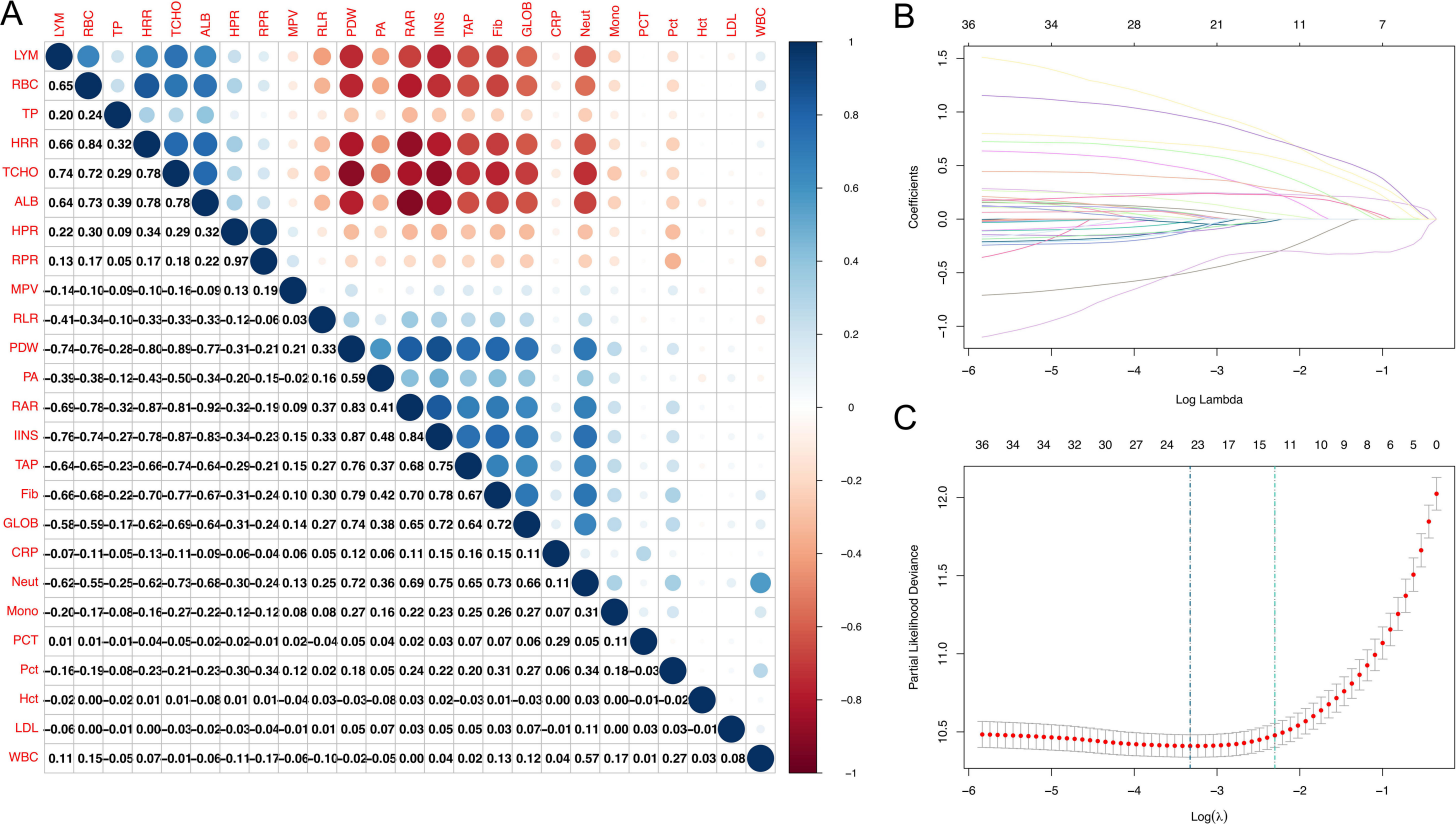
Determination of the number of factors by LASSO analysis and AUC comparisons between IINS and other variables. **(A)** A correlation matrix representing 25 indicators. **(B)** LASSO coefficient profiles of the 25 indicators. **(C)** Tenfold cross-validation for tuning parameter selection in the LASSO model.

**Figure 3:**
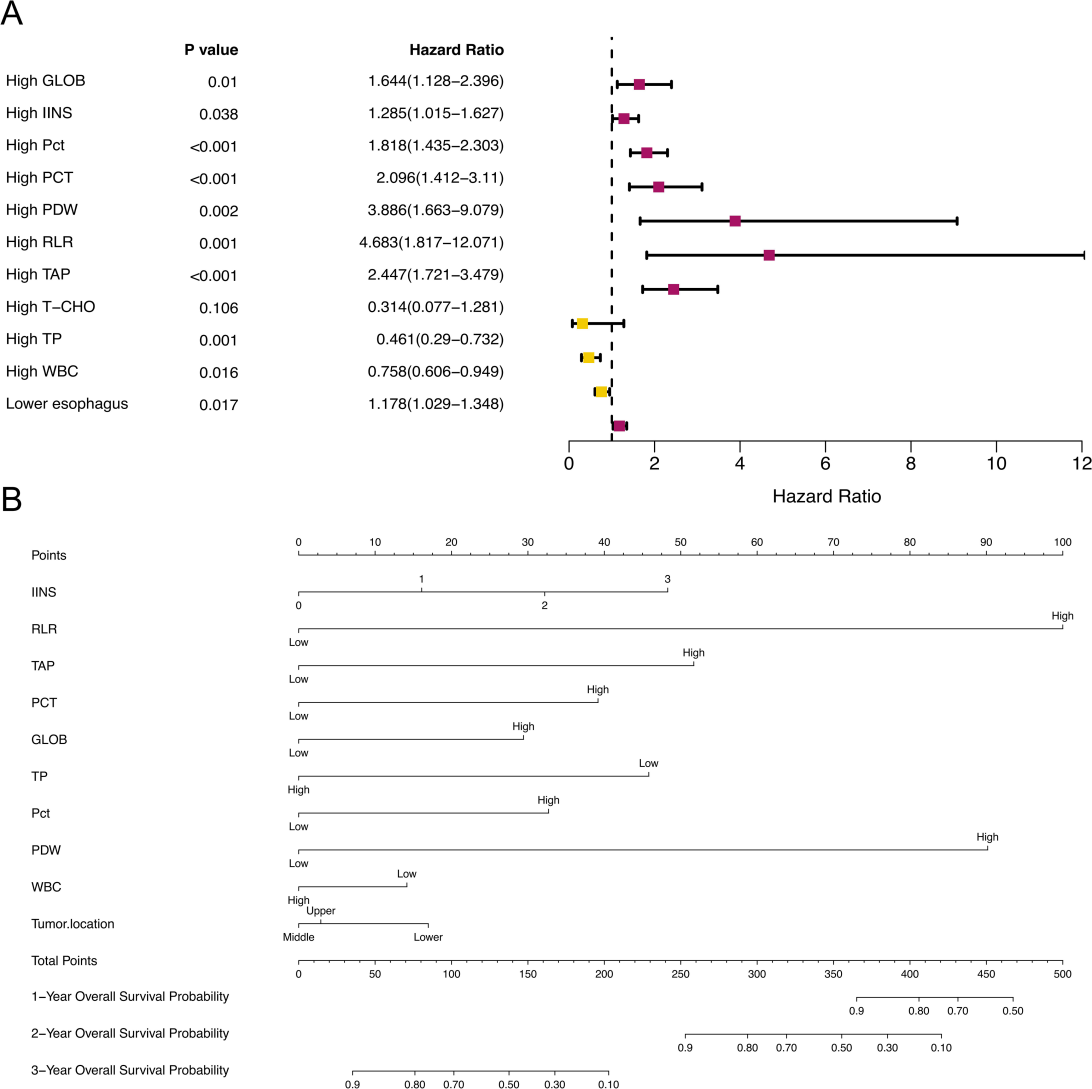
**(A)** Multivariate Cox regression analysis of EC based on IINS and RLR in the training cohort for OS. **(B)** Nomogram model of EC patients for predicting the 1-, 2-, and 3-year OS rates. To predict the 1-, 2-, and 3-year OS rates of EC patients, the patient’s IINS is located on the “IINS” axis. A straight line is drawn up to the “point” axis to determine the points for “IINS”. The process is repeated for each of the remaining axes, and a straight line is drawn each time to the “point” axis. The points received from each variable are added, and this point is located on the “total point” axis. A straight line is drawn down from the “total point” axis to the “1-year OS”, “2-year OS”, and “3-year OS” axes to determine the 1-, 2-, and 3-year OS rates of EC patients.

In **Figure 2a**, a correlation heatmap is shown for 6 nutrition and inflammation indicators and 19 laboratory indicators in 660 EC patients. We gradually selected independent factors affecting EC prognosis by LASSO univariate and multivariate Cox regression analyses. First, 24 relevant indicators were obtained through LASSO regression screening (**Figures 2b, c**) and input into the univariate Cox regression model. Variables with *p* < 0.10 were used for subsequent multivariate analysis. Finally, mixed regression, forward regression, reverse regression, and stepwise regression were used for regression analysis. The results of the variance analysis showed that the AIC values of the four models were roughly the same, and there was no statistically significant difference among the regression models (*p* ≥ 0.05). The final results show that GLOB (*p* = 0.010, 95% confidence interval [CI]: 1.128 ~ 2.396), IINS (*p* = 0.038, 95% CI: 1.015 ~ 1.627), Pct (*p* < 0.001, 95% CI: 1.435 ~ 2.303), PCT (*p* < 0.001, 95% CI: 1.412 ~ 3.110), PDW (*p* = 0.002, 95% CI: 1.663 ~ 9.079), TAP (*p* < 0.001, 95% CI: 1.721 ~ 3.479), TP (*p* = 0.001, 95% CI: 0.290 ~ 0.732), tumor location (*p* = 0.017, 95% CI: 1.029 ~ 1.348), WBC (*p* = 0.016, 95% CI: 0.606 ~ 0.949), and RLR (*p* = 0.001, 95% CI: 1.817 ~ 12.071) may be independent predictors of EC patients’ OS. In addition, we visualized the results in the form of forest maps (**Figure 3a**).

### 3.3 Nomogram establishment

Through the Cox regression model constructed sequentially in the previous step, 10 factors with significant effects on OS were obtained and incorporated into the nomogram to assess the 1-, 2-, and 3-year OS risk of EC (**Figure 3B**). According to the contribution of influencing factors to outcome variables, their value level was scored, and all scores were added to obtain the total score to calculate the predicted OS value. In general, the higher the total nomogram score, the greater the risk of recurrence. From the nomogram, we found that PLR, IINS, PDW, and TAP contributed the most to patient death.

### 3.4 Nomogram validation and evaluation

**Figure 4:**
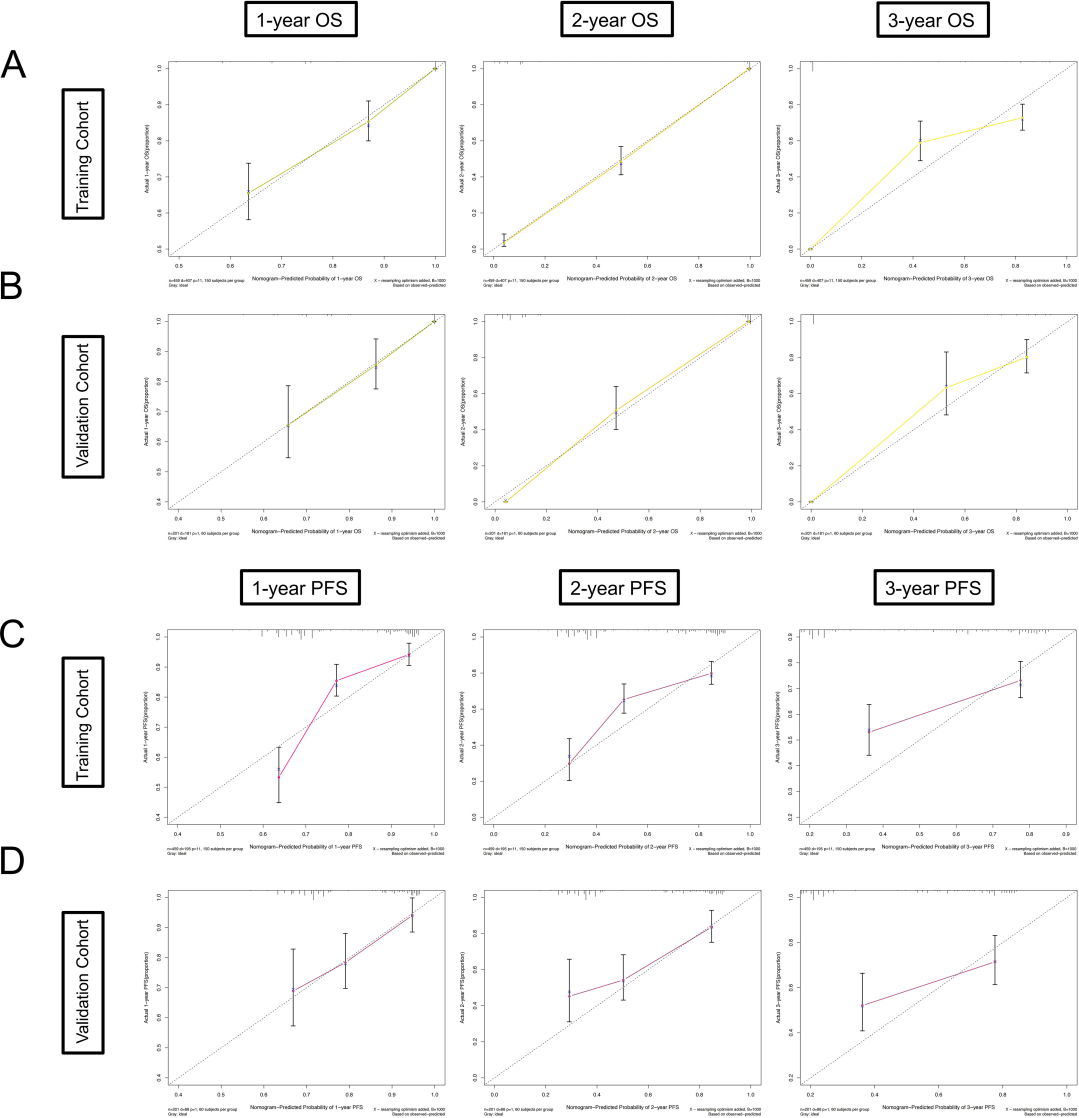
Calibration curves of the nomogram. **(A)** Calibration curves of the nomogram in the training cohort for 1-, 2-, and 3-year OS; **(B)** in the validation cohort for 1-, 2-, and 3-year OS; **(C)** in the training cohort for 1-, 2-, and 3-year PFS; **(D)** in the validation cohort for 1-, 2-, and 3-year PFS. The *x*-axis represents the model-predicted survival, and the *y*-axis represents actual survival. The bar represents 95% CI measured by Kaplan–Meier analysis, and the dotted line represents the ideal reference line.

**Figure 5:**
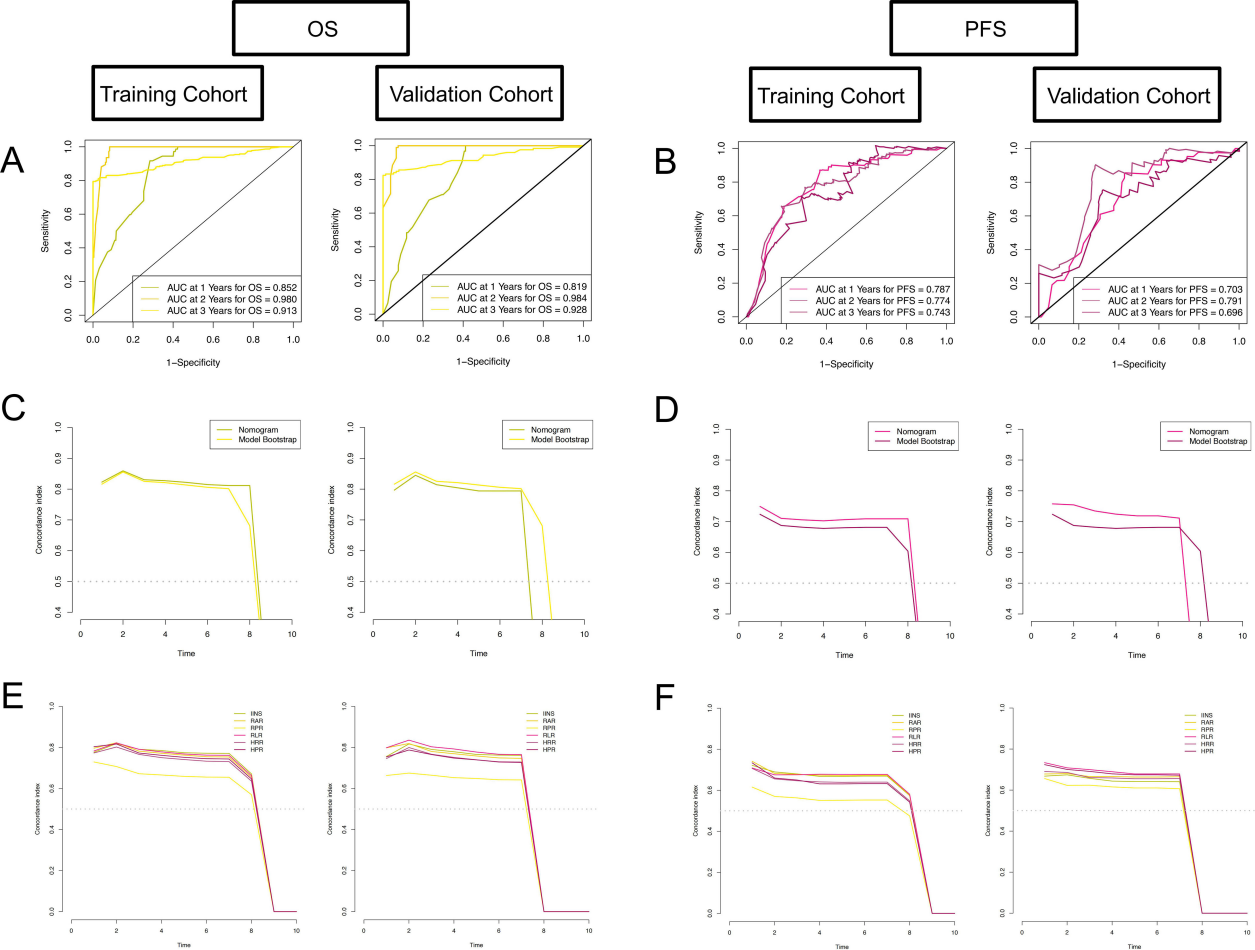
The prognostic performance of the nomogram and the prognostic performance of IINS, RAR, RPR, RLR, HRR, and HPR in patients with EC. The time-dependent ROC curves of the nomogram: **(A)** for OS in the training cohort and validation cohort; **(B)** for PFS in the training cohort and validation cohort. The time-dependent AUC curves of the nomogram: **(C)** for OS in the training cohort and validation cohort; **(D)** for PFS in the training cohort and validation cohort. The time-dependent AUC curves of IINS, RAR, RPR, RLR, HRR, and HPR: **(E)** for OS in the training cohort and validation cohort; **(F)** for PFS in the training cohort and validation cohort.

The C-index, time ROC, and time-dependent AUC were used to evaluate the discrimination of the nomogram. The C-indices in the training and validation cohorts were 0.824 and 0.798 based on OS and 0.721 and 0.677 based on PFS, respectively (**Table 4**). Through 1,000 repeated self-samplings for internal verification, the calibration curve shows that it is very close to the diagonal; that is, the predicted value is approximately equal to the actual value, so the comparison model prediction probability and observation probability are in good agreement (**Figure 4**). The AUCs in the training and validation cohorts were 0.852, 0.980, and 0.913, and 0.819, 0.984, and 0.928 (**Figure 5A**) for 1-, 2-, and 3-year OS, respectively, and those for PFS were 0.787, 0.774, and 0.743, and 0.703, 0.791, and 0.696 (**Figure 5B**), respectively. The time-dependent AUCs for predicting OS over 8 years were all > 0.8 (**Figure 5C**), and those for PFS were > 0.7 (**Figure 5D**), indicating favorable discrimination of the nomogram.

**Table 4:** C-index, NRI, and IDI of the nomogram and AJCC criteria-based tumor staging alone in survival prediction for EC patients.

Index	Training cohort			Validation cohort		
	Estimate	95% CI	*p*-value	Estimate	95% CI	*p*-value
C-index						
The nomogram for OS	0.824	0.808 ~ 0.840	~	0.798	0.772 ~ 0.823	~
The nomogram for PFS	0.721	0.688 ~ 0.754	~	0.677	0.624 ~ 0.730	~
The AJCC criteria-based tumor staging for OS	0.519	0.492 ~ 0.546	~	0.496	0.471 ~ 0.521	~
The AJCC criteria-based tumor staging for PFS	0.538	0.499 ~ 0.577	~	0.540	0.515 ~ 0.565	~
NRI (*vs.* the AJCC criteria-based tumor staging)						
For 1-year OS	0.505	0.329 ~ 0.611	~	0.503	0.001 ~ 0.824	~
For 2-year OS	0.894	0.851 ~ 1.240	~	0.925	0.000 ~ 1.419	~
For 3-year OS	0.605	0.510 ~ 0.750	~	0.809	0.499 ~ 0.888	~
For 1-year PFS	0.378	0.298 ~ 0.764	~	0.432	0.229 ~ 0.882	~
For 2-year PFS	0.460	0.293 ~ 0.824	~	0.592	0.177 ~ 1.279	~
For 3-year PFS	0.221	0.066 ~ 0.498	~	0.291	0.094 ~ 0.872	~
IDI (vs. the AJCC criteria-based tumor staging)						
For 1-year OS	0.202	0.133 ~ 0.268	< 0.001	0.182	0.093 ~ 0.305	< 0.001
For 2-year OS	0.824	0.754 ~ 0.863	< 0.001	0.848	0.723 ~ 0.899	< 0.001
For 3-year OS	0.581	0.493 ~ 0.645	< 0.001	0.670	0.526 ~ 0.756	< 0.001
For 1-year PFS	0.134	0.075 ~ 0.190	< 0.001	0.177	0.087 ~ 0.294	< 0.001
For 2-year PFS	0.297	0.196 ~ 0.390	< 0.001	0.402	0.250 ~ 0.523	< 0.001
For 3-year PFS	0.305	0.225 ~ 0.398	< 0.001	0.383	0.256 ~ 0.527	< 0.001

### 3.5 Clinical value of the nomogram compared with AJCC criteria-based tumor staging

The C-indices of the nomogram were 0.824 in the training cohort and 0.798 in the validation cohort based on OS, and 0.721 in the training cohort and 0.677 in the validation cohort based on PFS, which were higher than those of the AJCC staging system alone (**Table 4**).

**Figure 6:**
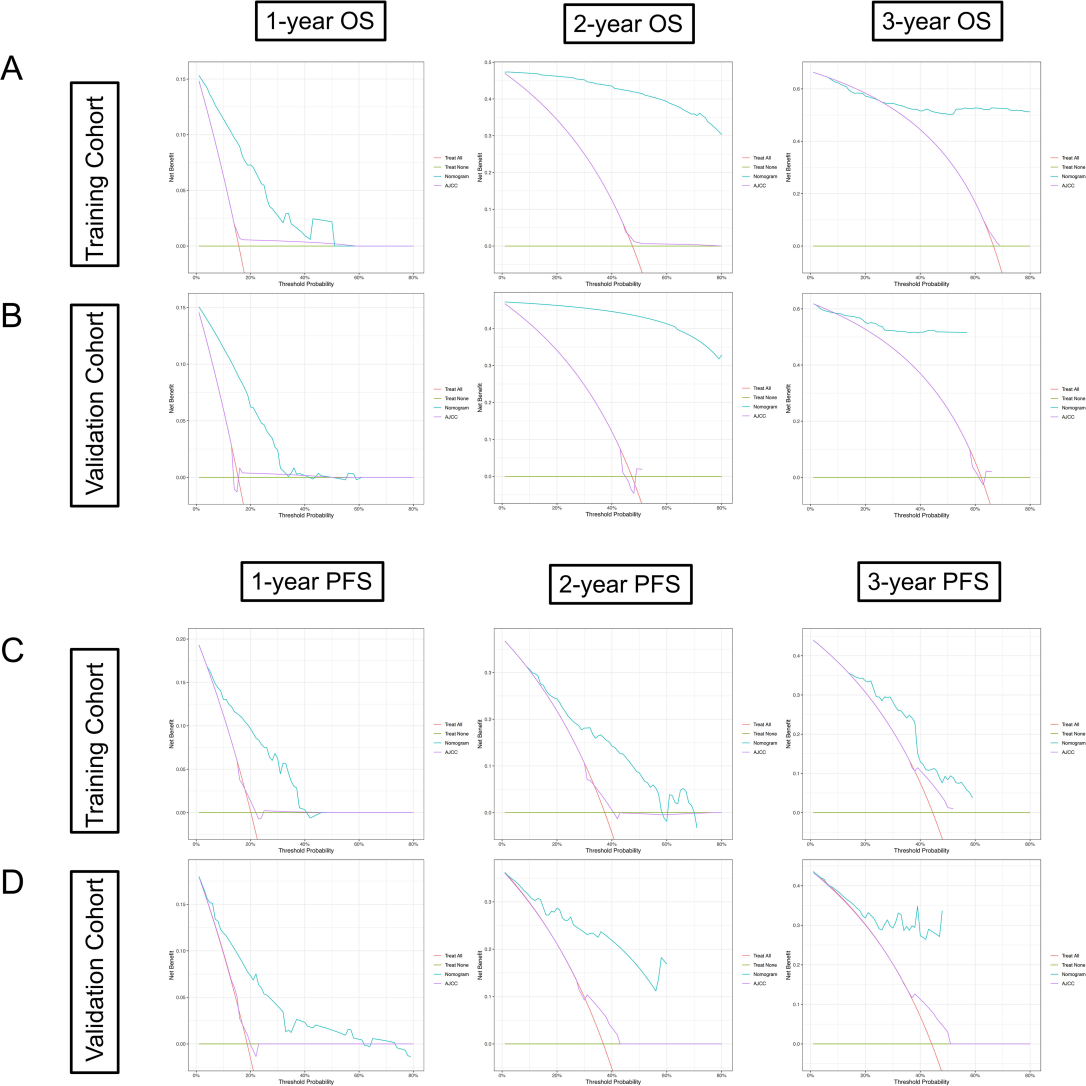
DCA curves of the nomogram and AJCC criteria-based tumor staging. The DCA curves were plotted based on **(A)** 1-, 2-, and 3-year OS benefit in the training cohort; **(B)** 1-, 2-, and 3-year OS benefit in the validation cohort; **(C)** 1-, 2-, and 3-year PFS benefit in the training cohort; **(D)** 1-, 2-, and 3-year PFS benefit in the validation cohort.

DCA was primarily used to determine which patients would benefit from the intervention. The nomogram’s net benefits were higher than those of treat all, treat none, and the AJCC staging system in both the training and validation cohorts, for OS and PFS, demonstrating that the nomogram is the optimal model with substantial net benefits in predicting 1-, 2-, and 3-year survival. It is worth using (**Figure 6**).

We then used the NRI and IDI to evaluate the improvement of our nomogram over the AJCC staging system. The results showed that regardless of the NRI or IDI, the results of OS prediction for 1-, 2-, and 3-year OS in the training cohort and validation cohort were all greater than 0. Notably, for 2-year OS, the NRIs were 0.894 and 0.925 in the training and validation cohorts and 0.460 and 0.592 for PFS, respectively. Similarly, the IDIs were 0.824 and 0.848 in the training and validation cohorts, respectively, and 0.297 and 0.402 for PFS, respectively, suggesting that the nomogram improved its ability to correctly reassign populations to different risk levels and its positive prediction probability for populations compared with the AJCC staging system (**Table 4**).

### 3.6 Survival analyses

**Figure 7:**
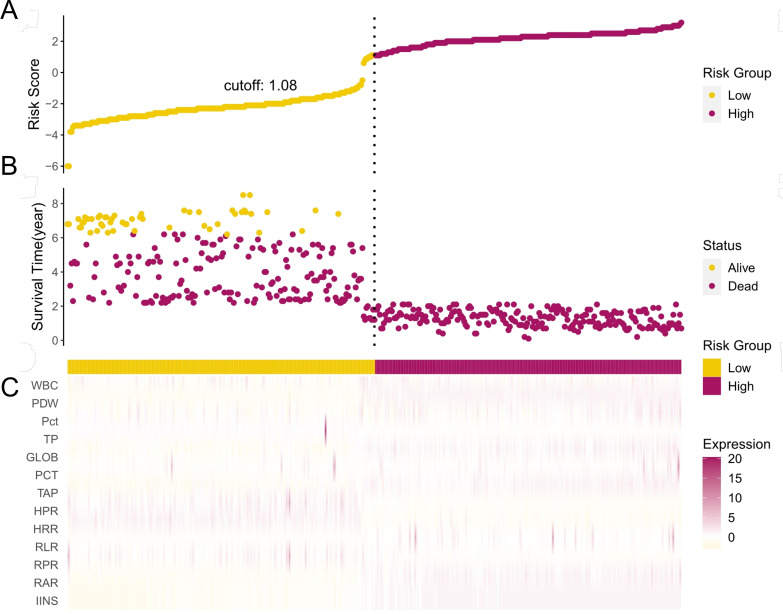
Relationship between the survival status/risk score rank and survival time (year)/risk score rank and the prognostic performance of IINS, RAR, RPR, RLR, HRR, and HPR in patients with EC. **(A)** The distribution of the risk score. **(B)** The survival duration and status of EC patients. **(C)** A heatmap of IINS, RAR, RLR, RPR, HRR, and HPR in the classifier.

To further explore the relationship between OS as well as PFS and each individual variable after low-risk grouping in EC patients after RS, scatter plots and heatmaps were drawn (**Figure 7**). By drawing the time-dependent AUC curves of IINS, RAR, RPR, RLR, HRR, and HPR over 0–10 years, we found that in both the training and validation cohorts, the AUCs of OS and PFS within 7 years were all greater than 0.8 (**Figure 5e**) and 0.7 (**Figure 5f**). The detailed results of variable screening and multivariable selection are presented in **Supplementary Table 1**.

**Figure 8:**
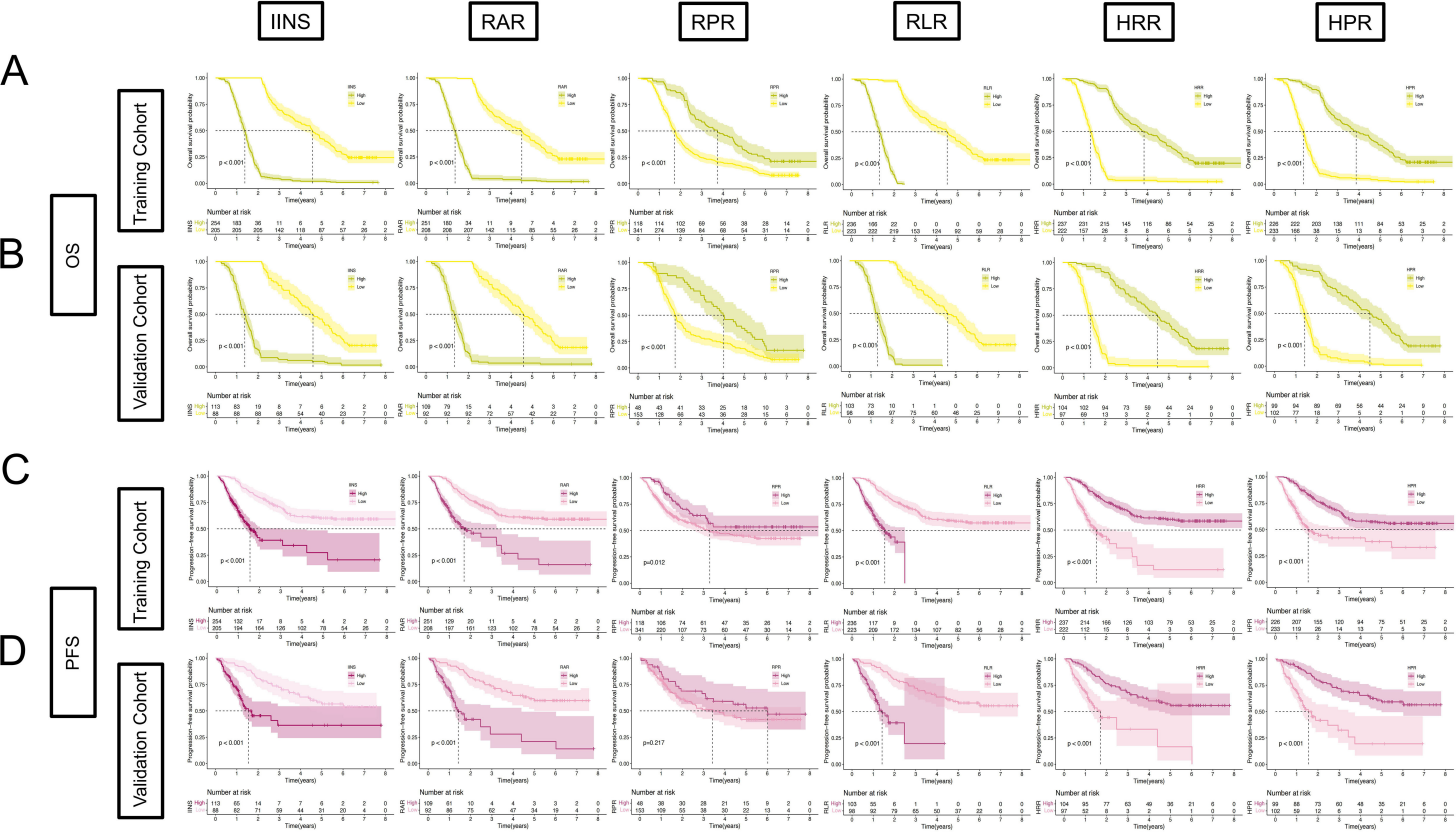
Kaplan–Meier curves for risk stratification. Kaplan–Meier plots for OS in the training cohort between **(A)** IINS, RAR, RPR, RLR, HRR, and HPR risk score groups; for OS in the validation cohort between **(B)** IINS, RAR, RPR, RLR, HRR, and HPR risk score groups; for PFS in the training cohort between **(C)** IINS, RAR, RPR, RLR, HRR, and HPR risk score groups; for PFS in the validation cohort between **(D)** IINS, RAR, RPR, RLR, HRR, and HPR risk score groups.

**Figure 9:**
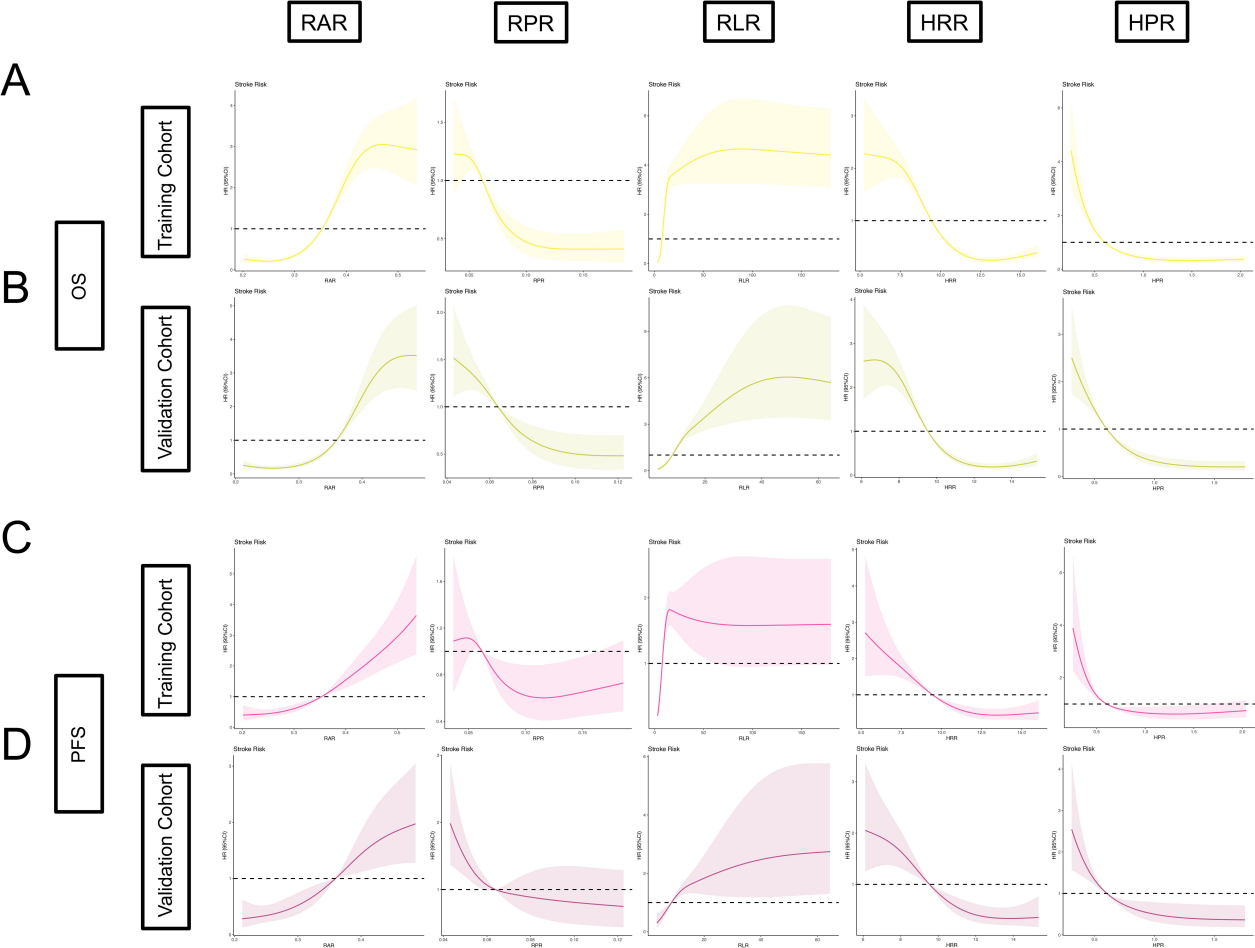
Association between RAR, RPR, RLR, HRR, HPR, and HR for OS and PFS using RCS regression models in patients with EC. **(A)** RAR, RPR, RLR, HRR, HPR, and OS in the training cohort; **(B)** RAR, RPR, RLR, HRR, HPR, and OS in the validation cohort; **(C)** RAR, RPR, RLR, HRR, HPR, and PFS in the training cohort; **(D)** RAR, RPR, RLR, HRR, HPR, and PFS in the validation cohort. (Unadjusted covariable).

**Figure 10:**
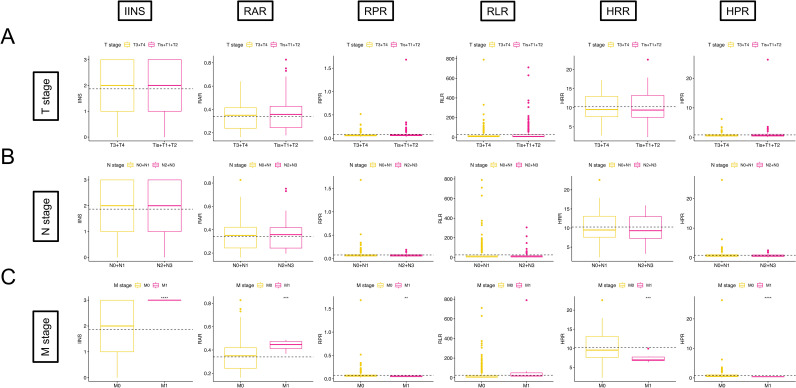
Relationship between TNM stage and nutrition and inflammation indicators. **(A)** T stage and IINS, RAR, RPR, RLR, HRR, and HPR. **(B)** N stage and IINS, RAR, RPR, RLR, HRR, and HPR. **(C)** M stage and IINS, RAR, RPR, RLR, HRR, and HPR.

In addition, by Kaplan–Meier and log-rank tests, we determined that higher IINS, RAR, and RLR were associated with poorer survival in EC patients, while RPR, HRR, and HPR were associated with more favorable survival, and the results were statistically significant (*p* < 0.001) (**Figure 8**). We further discuss the nonlinear relationship between these variables and survival risk in EC patients. The results showed that there was a significant nonlinear relationship between them as RCS variables and the HRs of OS and PFS (*p* < 0.001). With the increase in RAR and RLR, the survival risk of EC patients gradually increased, and with the increase in RPR, HRR, and HPR, the survival risk of patients gradually decreased (**Figure 9**). Finally, box diagrams were used to show the distribution of each nutrition and inflammation indicator with different TNM stages (**Figure 10**).

## 4 Discussion

With the progression of the study, we found that the factors affecting the survival of EC patients are complex and changeable and are related to stage, pathological type, lymph node metastasis, treatment methods, etc. Therefore, it is very important to find a prognostic evaluation criterion that can achieve accurate individualization. To date, the AJCC TNM staging system is the most commonly used system in the USA, including for EC. Accurate TNM staging is the most critical task for patients who are suitable for surgical treatment. However, the TNM staging system only includes tumor characteristics, such as tumor size (T), lymph node invasion (N), and metastatic status (M), and sometimes does not fully reflect prognosis. Therefore, in recent years, nomograms incorporating multiple prognostic factors in predicting cancer-related survival, recurrence, and metastasis have been extensively studied and have considerable potential. In this study, we constructed a new model to predict the OS of EC patients. Cox regression based on the minimum AIC selected 10 variables and included them in the nomogram. According to the standard deviation of the nomogram scale, RLR was the most important prognostic factor, followed by PDW, IINS, and TAP. The model validation indicated that it had favorable discrimination and calibration ability. Compared with the AJCC TNM staging system, both the NRI and IDI of the nomogram were positive, indicating that the predictive power of the nomogram was better than that of the AJCC staging alone. Furthermore, DCA demonstrated that our nomogram had better clinical application value and practicability in predicting OS. In addition, unlike previous studies on nomograms for EC patients, this study integrated IINS, RLR, and some clinically available classical factors and conducted internal validation, which is a rare and noteworthy innovation.

The prognosis of patients with digestive tract tumors is closely related to the nutritional status of the body and the strength of the immune system. Tumor cells multiply, invade, and metastasize by avoiding recognition by the body’s immune system, which is associated with 90% death rate of tumor patients (21–24). The IINS based on the combined preoperative CRP, LYM, and ALB scores has manifested good prognostic performance in hepatocellular carcinoma (25), endometrial cancer (26), and colorectal cancer (11). High IINS is usually caused by LYM, hypoproteinemia, and elevated CRP, indicating a high degree of inflammatory response, poor immune status, and poor nutritional status. Similarly, RLR based on preoperative RDW and LYM has also shown its predictive value in renal cell carcinoma (27), colorectal cancer (16), and hepatocellular carcinoma (28). For the first time, the prognostic significance of IINS and RLR in EC and their nonlinear relationships with HR were explored, and our results demonstrated that IINS and RLR promise to be independent predictors of recurrence in EC. We can also use the important value of these indicators to explain the predictive role of IINS and RLR on EC. The level of albumin reflects the anabolic function and reserve function of the liver, and the decrease in serum albumin is a sufficient indicator of the deterioration of clinical status, which is associated with the proliferation of tumor cells and the shortening of life expectancy (24). In cancer, LYM is a key mediator of humoral and cellular immunity, effectively suppressing tumor cells through different mechanisms, such as antibody-dependent cytotoxicity and complement activation. Its reduction may lead to immunosuppression or immune dysfunction, resulting in the loss of the body’s monitoring and elimination of malignant tumor cells and increased susceptibility to tumors (29–31). Inflammation is positively correlated with RDW (32–34), and persistent intratumoral inflammation leads to increased RDW.

Soviet scholars Kostyantin and Galakhin found that cancer cells can release abnormal glycoproteins and calcium-histone complexes in the metabolic process, and TAP is formed by the abnormal glycosylation of the protein–glycoprotein sugar chain structure or glycosyltransferase activity changes, so the appearance of TAP indirectly indicates the number and degree of cancer cells (35). TAP has high sensitivity and specificity for the detection of precancerous lesions, such as pancreatic cancer (36), lung cancer (37), gastric cancer (38, 39), thyroid carcinoma (40), bladder cancer (41), and colon cancer (42), and is an important indicator to judge the prognosis of tumors. However, the role of TAP in the occurrence and development of esophageal squamous cell carcinoma (ESCC) remains unclear and needs further study. Furthermore, some studies have shown that the relationship between PDW level and cancer prognosis is unclear, but recent studies have also reported that poor prognosis of breast cancer, colorectal cancer, and laryngeal cancer is associated with PDW level (43–48). PDW is an index indicating the change in platelet size, which is a sensitive marker of volume change in the process of platelet activation, pseudopodia formation, and shape change. Platelet activation can release angiogenic regulatory proteins, accelerate tumorigenesis and malignant progression, and promote epithelial cell to mesenchymal cell transformation (49, 50). In addition, platelet activation participates in the inflammatory response, resulting in leukocytosis, thrombocytosis, neutrophils, and lymphocytopenia. TAP and PDW deserve more attention, given their importance in our findings.

Notably, although RAR, RPR, HRR, and HPR were not independent prognostic factors in EC patients in our study, and these indicators were not included in our nomogram model, to our knowledge, few previous studies have comprehensively discussed their relationships with OS and PFS in EC patients, and we observed that RAR, RPR, HRR, and HPR had nonlinear relationships with OS/PFS HR. Between 0.30 and 0.45, the HR of RAR increased rapidly and then remained stable or decreased slightly. In contrast, the HR of RPR decreased rapidly from 0.05 to 0.10 and then remained stable or increased slightly. From 0 to 15, the risk of death and recurrence of RLR increased almost linearly and then entered a plateau. HRR was similar to HPR in that, as they increased, HR decreased accordingly. Therefore, our prognostic model needs to be further extended and can be used as a transitional tool for future studies.

TNM staging of EC is one of the important clinical characteristics of patients with EC and has an important impact on the choice of treatment and operation mode, as well as the prognosis of patients with EC. However, TNM stage was not an independent prognostic factor in the present study. The possible reason is that there are some differences in the staging of EC in different countries. In this study, we selected patients diagnosed with EC in the AJCC, Seventh Edition staging group for study. When we extend the results of this study to other countries or regions for cross-sectional comparisons, the EC stage needs to be redefined. The current TNM staging system has some limitations, which cannot achieve an individualized, accurate assessment in predicting the prognosis of patients. Nomograms are necessary aspects of modern medical decision-making (51–53). Therefore, we designed a quantitative model that integrates various factors, such as demographic and clinicopathological characteristics, to identify high-risk patients, which can be used as a complementary version of AJCC TNM staging to predict prognosis and guide clinical decision making. Despite the good performance of the nomogram we built, we still need to understand its limitations. First, this study is a retrospective cohort study, so it is difficult to avoid confounding factors and deviation of research results. Selection bias cannot be fully excluded due to the retrospective design and single-center cohort, although multivariable adjustment and balanced baseline characteristics between training and validation cohorts were applied to mitigate this risk. Second, the accuracy of the nomogram may be further improved by including nutrition and inflammation indicators measured at multiple time points before and after treatment. Third, this database was obtained from the Chinese Henan Province and might not represent all EC patients in China. Thus, larger multicenter studies should be performed for further validation. Although ROC-derived thresholds demonstrated good discrimination in this cohort and were supported by restricted cubic spline analyses, external validation is required to confirm their generalizability across different populations. We hope that our prediction model will be applied to various institutions and all sorts of cancer types and even uncertain malignancies in the future, and modify and improve it so that the design philosophy becomes more perfect and our prediction model becomes more accurate. Despite these limitations, our nomogram for surgical EC patients still has good model performance and clinical practical value, which can facilitate the evaluation of patients’ conditions, predict prognosis, and guide the clinical practice of EC patients.

## 5 Conclusions

Our nomogram model combining traditional predictors with IINS and RLR has higher accuracy and validity than the conventional AJCC staging system. The results of this study can be used for patient consultation, convenient prognostic assessment, and individualized follow-up strategies. Furthermore, this study provides enlightenment for us to encourage doctors to provide more individualized treatment for patients and reduce the burden on patients.

## Data Availability

The raw data supporting the conclusions of this article will be made available by the authors, without undue reservation.
